# Distribution of Geochemical Fractions of Phosphorus in Surface Sediment in Daya Bay, China

**DOI:** 10.3390/ijerph17124430

**Published:** 2020-06-19

**Authors:** Hongping Liao, Ciguang Pan, Lian Gan, Zhixin Ke, Huijuan Tang

**Affiliations:** 1 Joint Laboratory of Guangdong Province and Hong Kong Region on Marine Bio-Resource Conservation and Exploitation, South China Agricultural University, Guangzhou 510642, China; 20186140006@stu.scau.edu.cn (H.L.); 20192067003@stu.scau.edu.cn (C.P.); ganlian@scau.edu.cn (L.G.); 2 College of Marine Sciences, South China Agricultural University, Guangzhou 510642, China; 3 Key Laboratory of Tropical Marine Bio-resources and Ecology, South China Sea Institute of Oceanology, Chinese Academy of Sciences, Guangzhou 510301, China; kzx@scsio.ac.cn

**Keywords:** fractions of phosphorus, surface sediment, distribution, Daya Bay

## Abstract

Surface sediment samples were collected from 19 sites throughout Daya Bay, China, to study the concentrations, and spatial distributions of different fractions of phosphorus through sequential extraction methods. Like many coastal and marine areas, De-P was the dominant form of P, contributing 47.5% of TP, followed by O-P, contributing 25.5% of TP. Ex-P and Fe-P contribute the lowest to TP. The concentration of sedimentary TP ranged from 290.3~525.1 µg/g, with the average of 395.3 µg/g, which was a similar range to other estuaries and coastal areas. Based on the spatial distribution, Pearson correlation and Principal component analysis, different fractions of phosphorus showed different spatial distributions due to different sources. The molar ratio of organic carbon to phosphorus (TOC/O-P) ranged from 199 to 609, with the average of 413, which was much higher than the Redfield ratio, suggesting terrestrial sources of organic matter in Daya Bay surface sediment. The average bioavailable phosphorus was 149.6 µg/g and contributed 37.8% (24.6~56.0%) of TP, indicating that the surface sediments of Day Bay act as an important internal source of P.

## 1. Introduction

Phosphorus (P) is one of the most significant macronutrients in aquatic ecosystem. It plays an important role in phytoplankton growth, controls the primary production, and further influences aquatic biological structures and functions [1,2]. Naturally, the aquatic ecosystems are inclined to be P limited, due to their relatively low content and high requirement [3].

Estuaries and coastal bays are regions of land–ocean interactions. Increased inputs of nutrient from continental sources due to dense human population and intense land use have resulted in degradation of the coastal ecosystems [4]. When entering the coastal environment, only minimal P is biologically recycled within the water column, and most is finally settled and combined with sediment due to physical, geochemical and biological processes. However, incorporated sediment P can be released under appropriate pH, and redox potential conditions, etc [5]. Therefore, sediment acts as an important sink or source for P cycling [6]. When external inputs are controlled, P released from the sediments will still adversely affects the water quality in the long term [7]. The mobility of sediment P mainly depends on the composition of different fractions of P [8]. Research on the composition and distribution of P fractions in sediment is important for the determination of the risk of P release and to anticipate the potential effects on aquatic ecosystems.

Daya Bay is situated in one of the most rapidly developing areas in southern China. Industries including two nuclear power stations, stevedoring and petrochemical industries, printing and plastic factories have arisen around the coastal area in recent decades [9]. Furthermore, from 1987 to 2010, marine cage aquaculture had expanded from 142.5 to 1600 t [10]. Simultaneously, tourism and urbanization also rapidly developed [11]. As a result of these strong heavy anthropogenic activities, Daya Bay has frequently experienced severe eutrophication and harmful algal blooms (HAB) [12]. Moreover, increased human activity has altered the balance of nutrients [4,13]. Based on the long-term ratio of dissolved inorganic nitrogen (DIN) to phosphate, phytoplankton in Daya Bay shifted from nitrogen-limited to phosphate-limited since the mid-1990s [4], which has been attributed to increased external nitrogen loading and decreased external P loading. However, research on benthic fluxes of nutrient found positive diffusion of DIN and phosphate from sediment to overlying water, indicating sediment as an important source of this nutrient [7]. Daya Bay was one of the most intensively researched coastal areas in China, the sediment contamination also received considerable attention. However, most of the sediment researches are mainly focused on heavy metals [8,14,15] and persistent organic pollutants [16,17]. Compared with research on the above pollutants and research on other coastal areas [2,18], information on geochemical P fraction in the sediments of Daya Bay is rather limited, especially when the ecosystem was considered to be P-limited [4,13]. Studies on sediment P in Daya Bay are important to know the balance and the circulation of P in the ecosystem.

In this study, the content of different fractions of P, their spatial distribution, factors influencing their distributions, and the content of total nitrogen and total organic carbon were analyzed for further understanding of the current P levels and sources of sediment P in Daya Bay ecosystems.

## 2. Materials and Methods

### 2.1. Study Sites

**Figure 1:**
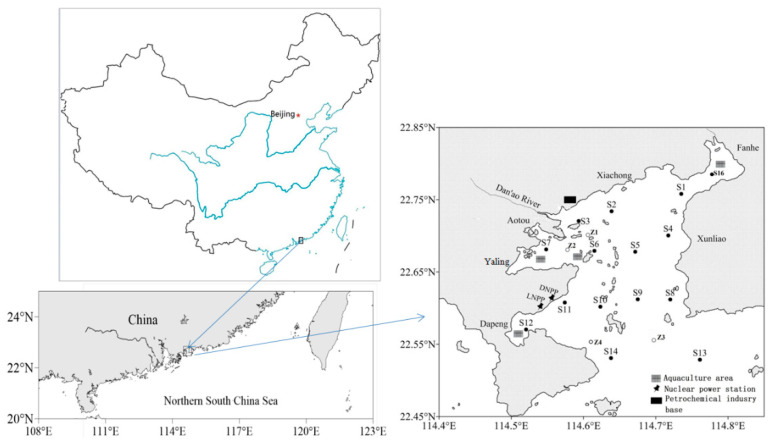
Daya Bay location and sampling sites (Labeled as S1-S14, S16, and Z1-Z4).

Located in the northern part of the South China Sea, the semi-enclosed Daya Bay has an area approximately 600 km^2^ (Figure 1). It is about 15 km from west to east and 30 km from north to south. Aotou and Yaling Bay, Fanhe Harbor, and Dapeng Cove are the main sub-basins in Daya Bay. The Aotou and Yaling sub-basins are in the northwest of Daya Bay, and functions as a busy port and cage aquaculture basis. Located in the northeast, Fanhe Harbor is a base of shellfish aquaculture and pond aquaculture around the bay, and Dapeng Cove, in the southwest, also has cage aquaculture and pond aquaculture nearby the coast. There is no major river discharge into the bay, except for the small Danao River in the northwest, so the water mainly originates from the South China Sea.

### 2.2. Sampling and Analysis

To represent the whole bay including the three sub-basins, north, west, central and outer bay, surface sediments of 19 sampling sites (Figure 1) were collected through a stainless sediment sampler in spring 2017. At the same time, depth, bottom temperature (Temp), asalinity and redox potential (Eh) were measured in situ through a YSI 6600 multi-probe sensor (Yellow Springs Instrument Co., Yellow Springs, OH, USA). Undisturbed surface sediments (top 5 cm layer) were placed in polypropylene bags and preserved at −20 °C. Then, the frozen sediments were freeze-dried, homogenized, grounded and sieved with a nylon 60-mesh (245 µm in diameter). The sieved samples were then kept in polypropylene bags until further analysis. Total nitrogen (TN) and total organic carbon (TOC) were measured through an elemental analyzer (FlashEA, 1112HT, Thermo Electron, Italy). Prior to analysis of TOC, inorganic carbon in each subsample was removed with 1M HCl for 3 h [19].

### 2.3. Extraction Method for Phosphorus

Sequential extraction procedures (SEDEX) [20] were used to determine different fractions of P. According to this method, five sedimentary P reservoirs are defined: exchangeable P (Ex-P), iron-bound P (Fe-P), authigenic P (Ca-P), detrital apatite P (De-P) and organic P (O-P). Inorganic P (I-P) in sediments was calculated as the sum of four forms excluding O-P. Total P (TP) was the sum of all the five forms. For each subsample, about 0.5 g sieved sediments was conducted according to the step-wise procedures of Ruttenberg [20] and Bastami et al. [21] (Table 1). After each step, the extraction was centrifuged, and the supernatant analyzed for phosphates through spectrophotometric phosphomolybdate blue method.

### 2.4. Statistical Analysis

Statistical analysis was conducted with IBM SPSS Statistics 22.0 (IBM, Armonk, NY, USA). The normality of the data was tested using the Shapiro-Wilk Test. When the data were normally distributed (*p <* 0.05), Pearson correlation (PC) analysis was performed to determine correlations between the different forms of phosphorus, TN, TOC, Temp, Eh and Salinity. Principle component analysis (PCA) was also applied to determine the relationships among different forms of phosphorus.

## 3. Results

### 3.1. Physiochemical Properties of Surfaces Sediments

**Figure 2:**
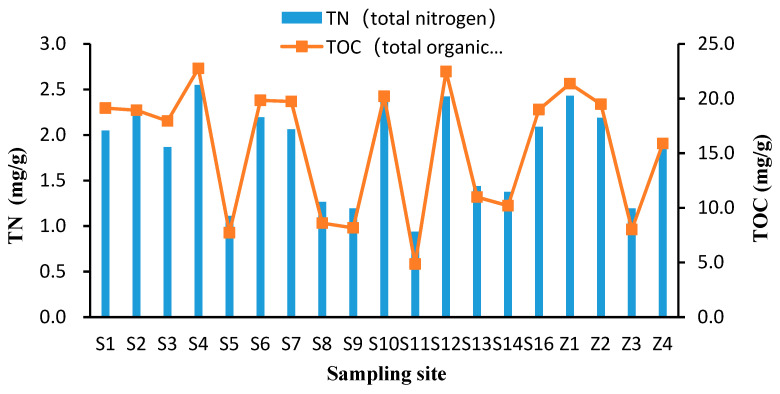
Dynamics of total nitrogen (TN) and total organic carbon (TOC) in the surface sediments of Daya Bay, China.

The depth of different sites of Daya Bay ranged between 3.3 and 20.0 m, with an average of 10 m. Sediment temperature during the sampling period ranged between 20.4 to 24.9 °C. Bottom salinity and Eh ranged from 31.0 to 34.4 and −61.0 to −356.0 mv, respectively. TN and TOC contents ranged from 0.94 to 2.55 and 4.86 to 22.76 mg/g, respectively (Table 2). The spatial distribution of TN and TOC contents were highly coincident, with the highest contents at site S4 and S12, and the lowest at sites S11, S8 and S9 (Figure 2).

### 3.2. Spatial Distribution of P Species in Surface Sediments

**Figure 3:**
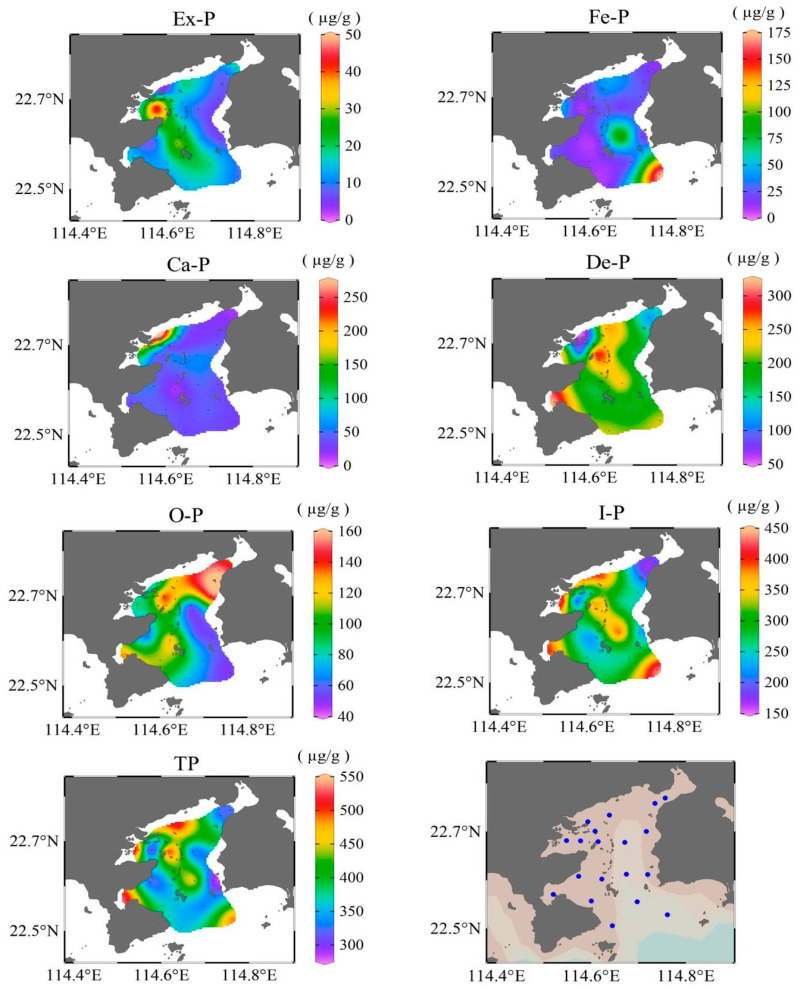
Spatial distributions of different P fractions in the sediment of Daya Bay, China.

**Figure 4:**
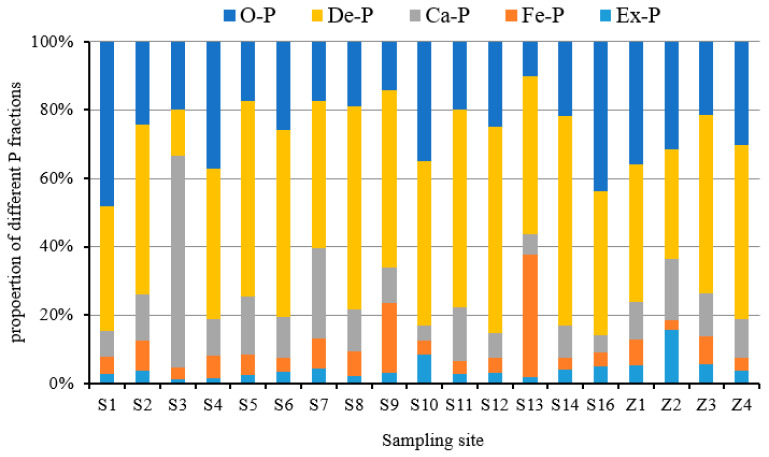
Composition of different P fractions in the surface sediment of Daya Bay, China.

In surface sediment of Daya Bay, the concentrations of different P species and TP present quite different spatial distributions (Figure 3). Ex-P content ranged from 4.49 to 45.4 µg/g, with an average concentration of 15.67 µg/g. The relative contribution of Ex-P to TP ranged from 1.01 to 15.6% (Figure 4), with the highest content at the mouth of Aotou Bay, followed by the central island site. The concentration of Fe-P ranged from 8.4 to 172.0 µg/g, with an average of 33.2 µg/g. Its relative contribution ranged from 2.9~35.7%, with the highest content and contribution at the outer bay site, followed by the central bay site. Ca-P content ranged from 15.2~274.3 µg/g, with an average concentration of 57.9 µg/g. Its relative contribution ranged from 3.3~62.0%, with the highest content and contribution in the northwest, where the Danao River flows in. Ca-P in most other areas was quite evenly distributed. De-P content ranged from 59.0 to 318.2 µg/g, with an average concentration of 187.8 µg/g. Its relative contribution ranged from 13.3~61.5 %; the highest content and contribution occurred at the mouth of Dapeng cove, and the lowest value occurred at the northwest site where the Danao River flows in. O-P content ranged from49.3~156.5 µg/g, with the average concentration of 100.7 µg/g. Its relative contribution ranged from 10.2~48.2%, with the highest content and contribution north-east of the bay. I-P content, which includes the sum of the former four species of P, contributes 51.0~89.8% of the TP. TP content ranged from 290.3~525.1 µg/g with the average concentration of 395.3 µg/g. TP content was relatively higher in Yaling Bay, Dapeng Cove, north coastal area, and the outer bay site. On average, the percentage of different P forms relative to TP were De-P (47.5%) > O-P (25.5%) > Ca-P (14.7%) > Fe-P (8.4%) > Ex-P (4.0%).

### 3.3. Correlation and Principal Component Analysis

**Figure 5:**
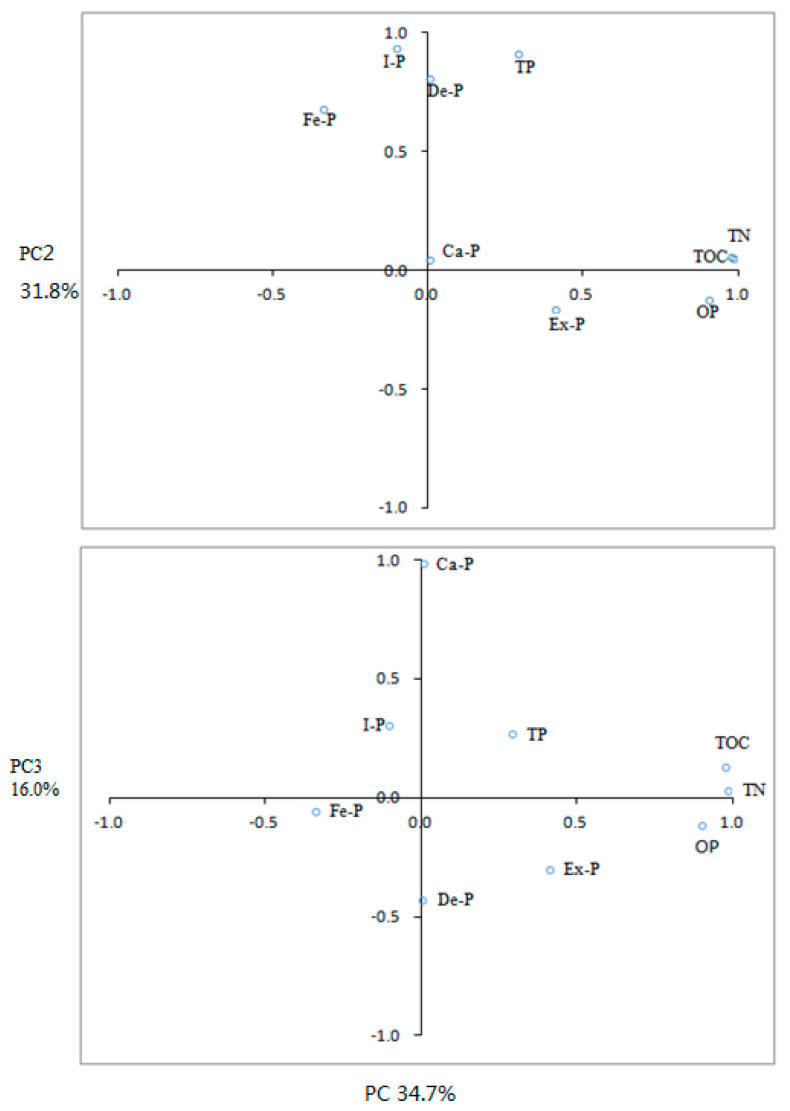
PCA loadings (PC1 versus PC2 and PC3) for different P fractions, TN and TOC in surface sediments.

Pearson correlations among different P species, TP, TN and TOC, were analyzed (Table 3). Ex-P did not significantly correlate with any other indicators (*p >* 0.05). Both Fe-P and De-P were significantly correlated with I-P and TP (*p* < 0.05), and O-P, TN and TOC showed a positive and significant correlation with each other (*p* < 0.01). Principle component analysis is widely used to verify and quantify pollution sources. Based on PCA, three components (PC1, PC2 and PC3) accounting for 82.5% of the total variance were extracted (Figure 5). PC1 had high positive loadings on variables TN, TOC and O-P, accounting for 34.7% of the total variance. PC2 had high loadings for variables TP, I-P, De-P, and Fe-P, accounting for 31.8% of the total variance. PC3 showed high loading for Ca-P, accounting for 16.0% of the total variance.

### 3.4. Sedimentary TOC to O-P

**Figure 6:**
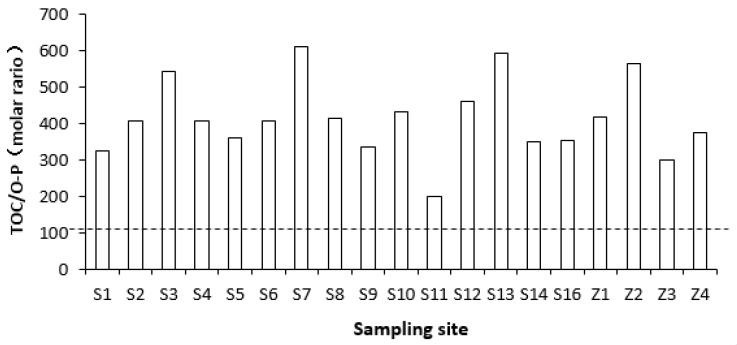
Atomic ratios of TOC and O-P in surface sediments of Daya Bay.

The TOC/O-P ratio ranged from 199 to 609, with an average of 413 (Figure 6). The lowest value occurred at the site near the nuclear power stations.

## 4. Discussion

### 4.1. Phosphorus species

Ex-P is formed as phosphate adsorbs directly onto the surface of sediments minerals [22], and therefore it can be readily released and become available to phytoplankton [18]. In the present research, Ex-P contribute the lowest to TP (4%), as was also found in Jiaozhou Bay [23], the East China Sea Shelf [24], Maowei Sea in the Beibu gulf [25], and Changjiang Estuary [26]. The average concentration (15 µg/g) was also in a similar range to these estuaries and bays (Table 4), but it was much lower than that in South China Sea [27], south Long Island (USA) [28], Kalpakkam( India) [29], southern Caspian Sea [21], and the eastern coast of Hainan Island [30] (Table 4). Relatively high values of Ex-P were generally related to a high input of phosphate from the rivers or coastal sewage, such as in the Pearl River, Changjiang River, and Wanquan Rive [27,30,31,32]. The research indicates that organic matter may also be an important factor regulating the Ex-P concentration because of the high correlation of Ex-P with TOC contents [2,23]. However, Ex-P in Daya Bay was not significantly correlated with TOC or other parameters (Table 3). Exceptionally high values occurred at the mouth of Aotou Bay, but may be related to a combination of urban sewage and cage aquaculture. [14].

Fe-P can release phosphate with the decrease in environmental redox or under anoxic conditions [7,8]. In the present research, Fe-P contributed the second lowest to TP, as was also found in sediments of Hainan coastal area [30], East China Sea Shelf [24], Yellow Sea [35] and East China Sea [32] and the southern Caspian Sea [21]. The average concentration of Fe-P in Daya Bay (33.2 µg/g)was comparable to that in Zhangzi Island [18], South China Sea [2] and Hainan Island [30], higher than East China sea shelf, Little Maderia Bay, Changjiang estuary [24,26,35], and lower than that in Pearl River Delta [33], Laizhou Bay [18], Kalpakkam of India [29], Maowei Sea in Beibu Gulf [25], southern Caspian Sea [21] and the central Pacific Ocean [1] (Table 4). Many studies suggested that Fe-P decreases from brackish to saline water (relative high salinity) [31]. Conversely, the salinity of Daya Bay increased slowly from the inner to outer bay [13], but the highest Fe-P value occurred at the outer bay site, which should have relatively higher salinity and be less polluted. A more oxidized environment at the outer bay might shift soluble Fe (II) to particulate Fe (III) and enhance the adsorption of phosphate onto Fe oxides/hydroxides, thus increasing the content of Fe-P. This was supported by a negative correlation between Fe-P and O-P, though the significance was not reached (r = −0.394, *p* = 0.095), indicating the need for further investigation.

Ca-P mainly indicates phosphorus incorporated with CaCO_3_, including biogenic apatite (bones, teeth, shell fragments, and calcareous phytoplankton, etc.), and authigenic carbonate fluorapatite (CFA) [30,31]. Averaged Ca-P concentration in Daya Bay was 57.92 µg/g, which was relatively lower than in many other coastal and marine areas (Table 4), but higher than Zhangzi Island, Maowei sea and, Changjiang Estuary [18,25,26]. The content of Ca-P was highest in the nearby Danao River estuary and decreased gradually into the bay, which suggested a riverine input of Ca-P PCA analysis and indicated that PC3 had high loading for Ca-P only, suggesting a different source of Ca-P with other P fractions.

De-P includes detrital apatite and other P-containing minerals, and was the most abundant P fraction in Daya Bay, accounting for 47.5% of TP, which was similar to studies in other coastal areas [24,33]. Previous research suggested that the eroded soils enriched in De-P from upper basins of rivers resulted in a high contribution of De-P, such as that in the Yangtze River Estuary and adjacent East China Sea inner shelf [26,31], the Bohai and Yellow Seas, and the Yellow River Estuary [35], and the Pearl River Delta [33]. In other estuaries, such as the Amazon Estuary, because of river flow through tropical rainforests, grass, and fertile soils, the contribution of De-P was only 6% of TP [1]. There are no major river flows, except the small Danao River in the northwest; the river had a high content of Ca-P and very low contribution of De-P (3.3%), indicating that riverine input of De-P was negligible. The highest content of De-P occurred at sites S6 and S12, indicating sources from cage aquaculture.

O-P contributed an average of 25% of TP, which was comparable to that in South China Sea (24.9%) [2], Hailan Island (25%) [30], and Bay of Seine (22%) [22], higher than that in Laizhou Bay, Zhanzi Island [18], East China Sea shelf [24], Kalpakkam [29] and southern Caspian Sea [21], but less than that in Pearl River Delta [35], Maowei Sea [25] and Changjiang Estuary [26]. The highest O-P concentration occurred at the northeast, around Fanhe bay, which was consistent with the surface sediment content of TOC and TN. Accordingly, O-P showed significant correlations with TOC (r = 0.856, *p* < 0.0001) and TN (r = 0.844, *p* < 0.0001). PCA analysis showed PC1 had high positive loadings on variables TN, TOC and O-P, suggesting similar sources of TN, TOC and OP. Significant positive correlations between TOC and O-P have been widely found in many other studies due to simultaneously bound TOC and O-P onto fine-grained sediments [2,18,23,29]. In addition, the higher O-P levels at Fanhe Bay were probably related to the discharge of intensive pond aquaculture effluents around the bay coast, which was also indicated by relatively higher chla at Fanhe Bay [13]. High phytoplankton biomass might also be deposited on the surface of sediment and result in a high content of TOC and O-P.

The average TP concentration of 395.3 µg/g (291~511 µg/g) in the surface sediment of Daya Bay was similar to other Chinese estuaries and bays (Table 4). In Daya Bay, there was a small TP increase compared with a previous average of 341.9 µg/kg [34] due to an increase in O-P, while total I-P remained relatively stable, although the composition of I-P differed. According to sediment quality guidelines (SQGs, Ministry of Environment and Energy, Ontario, Canada) [36], sediment TN and TP content below 550 and 600 ug/g, respectively, indicates low pollution. Therefore, Daya Bay had low phosphorus pollution, and moderate nitrogen pollution (4800 mg/kg, the severe pollution standard).

### 4.2. Sedimentary Organic Carbon to Phosphorus Ratio

The molar ratio of organic carbon to phosphorus (TOC/O-P) is widely used to determine the origin of sources of O-P and TOC [26,28]. According to Redfield ratio [3], the TOC/O-P of marine phytoplankton is generally close to 106 [37], so the organic matter in sediment originating from marine plankton sedimentation should be also around 106, while the ratio > 106 indicates land sources [21]. A lower value of the ratio may happen in the aerobic/suboxide areas with low TOC and/or dominance of bacterial biomass [28,37]. Due to different sources of terrestrial and marine organic matter, and/or preferential regeneration of organic carbon and phosphorus, TOC/TOP ratios in marine sediments range widely from 50 to 4500 [38,39]. TOC/O-P in the surface sediment of Daya Bay had a much higher value than Redfield ratio, indicating mainly terrestrial sources of organic matter, especially at sites S7, Z2 and S3, which were located in the busy harbor, aquaculture area and Danao River estuary, respectively. Another very high value was found at the most seaward site, S13, due to a very low content of O-P, which was significantly negatively correlated with salinity. The lowest value occurred adjacent to nuclear power stations (site 11), which had relatively lower content of TOC, most probably due to the higher decomposition of TOC at higher water temperatures caused by discharges of cooling water from the two nuclear power stations [40].

### 4.3. Bioavailable Forms of P Phosphorus

Bioavailable phosphorus (BAP) in sediment indicates the total amount of soluble phosphate that could be released directly and/or after conversion into the overlying water. Ex-P, Fe-P and O-P can be readily released [41], and the sum of the three fractions was considered as potential BAP. BAP concentration of surface sediments in Daya Bay ranged from 108 to 188 µg/g, with an average concentration of 149.6 µg/g, which contributed 37.8% (24.6~56.0%) of TP. The relatively high proportion was in similar range with that of the continental shelf region of the northern South China Sea (36.7%), eastern coast of Hainan Island (38.6%) and intertidal surface sediments of the YRE (37.4%) [2,30,31]. This suggests that surface sediments of Daya Bay may be an important bioavailable P resevoir. Recent benthic nutient flux investigation indicate sediment was a P source for phytoplankton in summer and autumn, and was a P sink in winter and spring in Daya Bay [42]. Due to a rapid increase in dissolved inorganic nitrogen and marked decreased phosphate during recent decades, Daya Bay was believed to shift from nitrogen to phosphorus limitation since the 1980s to the mid-1990s. However, the average chla content kept an increasing trend [4]. The release of P may play a role in supporting increased harmful algal blooms (HABs), which usually occur in the summer and autumn in Daya Bay [11,42]. Other research indicated that algal blooms may enhance the P release from sediment caused by pytoplankton exploitation of phosphate in the water column or mediated by physicochemical factors change coinciding with algal blooms [43]. However, further research on the interactions of phytoplankton with benthic P cycling in Daya Bay is needed.

To summarize, based on spatial distribution, PC and PCA analysis, sediment P in Daya bay is mainly derived from terrestrial sources, such as pond and cage aquaculture, riverine input and urban sewage. A high content of BAP in sediments indicates a high potential of phosphate supply to pelagic phytoplankton, especially the HABs. Cutting off the terrestrial sources of P in Daya Bay is essential for decreasing sediment phosphorus and the health of the ecosystem in the long term.

## 5. Conclusions

The concentration and spatial distribution of different fractions of P in surface sediment of Daya Bay were quantified through extraction methods. Like many coastal and marine areas, De-P was the dominant forms of P, contributing 47.5% of TP, followed by O-P, contributing 25.5%, while Ex-P and Fe-P contribute the lowest to TP. The concentration of sedimentary TP ranged from 290.3~525.1 µg/g with the average of 395.3 µg/g, which was in similar range to other estuaries and coasts. Different fractions of phosphorus showed different spatial distributions due to different sources, which were mainly derived from terrestrial sources, such as pond and cage aquaculture, riverine input and urban sewage. The molar ratio of organic carbon to phosphorus (TOC/O-P) ranged from 199 to 609, and was much higher than the Redfield ratio, suggesting terrestrial sources of organic matter in Daya Bay surface sediments. The average bioavailable phosphorus was 149.6µg/g and contributed 37.8% (24.6~56.0%) of TP, indicating that the surface sediments of Day Bay act as an important internal source of P.

## Figures and Tables

**Table 1:** Phosphorus fraction procedures.

Extraction Agent	Extraction Condition (25 °C)	P-Fraction
Step 1: 1 M MgCl_2_ (pH = 8.0).	Shaking for 2 h twice, wash with pure water for 2 h	E*x*-P
Step 2: 0.30 M Na-citrate 1.0 M NaHCO_3_ (pH 7.6), 1.125 g of Na-dithionite in 45 mL of citrate bicarbonate solution	Shaking for 8 h; wash with MgCl_2_ and pure water for 2 h respectively	Fe-P
Step 3: 1 M Na-acetate buffered to pH 4 with acetic acid	Shaking for 6 h, wash with MgCl_2_ and pure water for 2 h, respectively	Ca-P
Step 4: 1 M HCl	Shaking for 16 h	De-P
Step 5: Ash at 550 °C 1 M HCl	1 M HCl extraction for 16 h of residue ashed at 550 °C for 2 h	O-P

**Table 2:** Physiochemical properties of surfaces sediments of Daya Bay.

Items	Depth (m)	Salinity	Temp (°C)	Eh (mv)	TN (mg/g)	TOC (mg/g)
Mean	10.9	32.6	22.2	−175.2	1.84	15.54
Max	20.0	34.4	24.9	−61.0	2.55	22.76
Min	3.3	31.0	20.4	−356.0	0.94	4.86

**Table 3:** Pearson correlation (PC) coefficient matrix of different P fractions, total nitrogen (TN), and total organic content (TOC) in the surface sediments of Daya Bay.

	Ex-P	Fe-P	Ca-P	De-P	IP	O-P	TP	TN	TOC	Salinity	Temp	Eh
Ex-P	1											
Fe-P	−0.176	1										
Ca-P	−0.189	−0.09	1									
De-P	−0.094	0.306	−0.358	1								
IP	−0.169	0.614 **	0.369	0.632 **	1							
O-P	0.144	−0.394	−0.158	−0.029	−0.3	1						
TP	−0.112	0.463 *	0.313	0.643 **	0.906 **	0.132	1					
TN	0.385	−0.235	0.024	−0.008	−0.054	0.856 **	0.324	1				
TOC	0.349	−0.238	0.122	−0.047	−0.021	0.844 **	0.353	0.989 **	1			
Salinity	−0.014	0.422	−0.328	0.220	0.131	−0.705 **	−0.177	−0.650 **	−0.708 **	1		
Temp	−0.161	−0.016	0.533*	−0.338	0.094	0.409	0.280	0.394	0.471 *	−0.632 **	1	
Eh	−0.376	−0.042	−0.330	0.117	−0.207	−0.266	−0.333	−0.551 *	−0.574 *	0.359	−0.230	1

* Significant correlation at *p* < 0.05. ** Significant correlation at *p* < 0.01.

**Table 4:** The average concentration of different P forms in surface sediments determined in this study compared with measurements from other estuaries and coastal seas (μg/g)

Locations	Ex-P	Fe-P	Ca-P	De-P	O-P	I-P	TP	References
Daya Bay	15.7	33.2	57.9	187.8	100.7	294.5	395.3	The present study
Central Pacific Ocean	10.9	44.8	621.8	809.9	94.1		1581.4	[1]
South China Sea	43.2	34.9	127.8	154.1	115.2	345.8	461.0	[2]
Laizhou Bay	10.7	40.7	60.2	320.0	62.1	423.6	493.7	[18]
Zhangzi Island	13.1	36.6	31.4	145.4	49.4	226.0	275.6	[18]
Long Island Sound, USA	34.9	-	126.4	178.8	95.9	-	436.0	[20]
Southern Caspian Sea	46.4	73.5	158.2	177.9	79.6		535.8	[21]
Bay of Seine	25.3	42.5	186.4	-	75.5	-	329.0	[22]
East China Sea shelf (spring)	13.8	21.9	148.0	153.0	91.7	-	428.4	[24]
East China Sea shelf (autumn)	11.4	20.0	170.4	225.0	77.1	-	503.9	[24]
Maowei Sea, China(summer)	15.3	125.2	57.9	52.6	156.6		407.6	[25]
Maowei Sea, China(winter)	16.6	77.1	33.0	57.3	113.0		297.0	[25]
Changjiang Estuary and adjacent East China Sea inner shelf	14.0	13.7	29.4	302.4	183.4		542.9	[26]
Kalpakkam, India	63.6	58.3	737		138.0		997.0	[29]
Hailan island	29.9	36.1	131.9	159.1	119.0	356.9	475.9	[30]
Little Madeira Bay	2.3	<1	73.7	-	65.2	-	106.0	[32]
Pearl River Delta	-	79.7	30.5	222.6	167.0	334.0	501.0	[33]
Daya Bay	19.7	27.4	92.9	127.7	51.1	290.8	341.9	[34]

Notice: - no data available; some of the data were originally in µmol/g.
